# Temporal Interference (TI) Stimulation Boosts Functional Connectivity in Human Motor Cortex: A Comparison Study with Transcranial Direct Current Stimulation (tDCS)

**DOI:** 10.1155/2022/7605046

**Published:** 2022-01-31

**Authors:** Zhiqiang Zhu, Yiwu Xiong, Yun Chen, Yong Jiang, Zhenyu Qian, Jianqiang Lu, Yu Liu, Jie Zhuang

**Affiliations:** ^1^The Research Group of “The Effects of Non-Invasive Deep Brain Stimulation on Improving Human Performance and Its Mechanisms”, Shanghai University of Sport, Shanghai 200438, China; ^2^Faculty of Education of Shenzhen University, Shenzhen 518061, China; ^3^School of Psychology, Shanghai University of Sport, Shanghai 200438, China

## Abstract

Temporal interference (TI) could stimulate deep motor cortex and induce movement without affecting the overlying cortex in previous mouse studies. However, there is still lack of evidence on potential TI effects in human studies. To fill this gap, we collected resting-state functional magnetic resonance imaging data on 40 healthy young participants both before and during TI stimulation on the left primary motor cortex (M1). We also chose a widely used simulation approach (tDCS) as a baseline condition. In the stimulation session, participants were randomly allocated to 2 mA TI or tDCS for 20 minutes. We used a seed-based whole brain correlation analysis method to quantify the strength of functional connectivity among different brain regions. Our results showed that both TI and tDCS significantly boosted functional connection strength between M1 and secondary motor cortex (premotor cortex and supplementary motor cortex). This is the first time to demonstrate substantial stimulation effect of TI in the human brain.

## 1. Introduction

The neuromodulation effects of noninvasive brain stimulation technologies on neurorehabilitation have gained great attention in scientific and clinical communities. It has a significant effect and is widely used to optimize motor control, motor learning, and treat a motor-related neuropsychiatric disorder, such as Parkinson's disease and poststroke rehabilitation [1–5]. The prevalent noninvasive brain stimulation approaches include transcranial magnet stimulation (TMS), transcranial direct current stimulation (tDCS), and transcranial alternating current stimulation (tACS). For example, tDCS has been considered as a promising ergogenic potential method in neuromodulation [6–8]. A brand-new noninvasive neural stimulation method—temporal interference (TI)—was developed recently, but its practical effects still need to be tested widely and intensively before it is broadly accepted. To this end, here, we aim to investigate whether TI is effective in boosting the human motor cortex, compared with tDCS.

Previous studies have demonstrated that tDCS produced effective neuromodulation by applying a low-intensity current (1-2 mA) delivered to the scalp. The neuromodulation effects are highly polarity-dependent. Anodal tDCS functions increase cortical excitability in the primary motor cortex (M1), whereas cathodal tDCS leads to decreasing cortical excitability by alternating the resting membrane potential [9, 10]. In line with the modulation in cortical excitability, anodal tDCS stimulating M1 improved motor behavior in the contralateral hand, while cathodal tDCS stimulating M1 resulted in contralateral hand functionally ineffective [3, 11].

The functional magnetic resonance imagining (fMRI) technique provides critical insight in exploring the mechanisms of tDCS effects on brain function [11, 12]. Recent studies showed that tDCS increased resting-state functional connectivity during and post stimulation [13, 14]. Mondino and his colleagues [13] compared the neuromodulation effect of tDCS with the sham condition and found that tDCS increased resting-state functional connectivity between the left dorsolateral prefrontal cortex and bilateral parietal region. Moreover, Polania et al. [14] applied anodal tDCS to each individual's left M1 and found that the functional connectivity was enhanced between the left somatomotor cortex (SM1) and premotor and superior parietal region. These functional connectivity changes induced by tDCS might be related to neuroplastic alteration of relevant brain regions. In addition, the study of Sehm et al. [11] suggested that tDCS might modulate both intracortical and interhemispheric connections with M1 using a seed-based analysis. However, the current tDCS techniques are limited to low-intensity current by the safety guideline [15, 16] and cannot focally stimulate deeper brain regions.

To solve this issue, Grossman and his colleagues discovered a new noninvasive neural stimulation strategy, called “temporal interference (TI).” It could achieve much deeper and focal stimulation without affecting adjacent brain regions. This strategy applied two channels with slightly different high-frequency alternating currents. The frequency of single channel is too high to activate neural firing, but the frequency of the envelope electrical field generated by two channels is lower enough to focally activate neural activity. For instance, Grossman and colleagues applied 2 kHz and 2.01 kHz stimuli on the motor cortex of mice. The two channels generated a low-frequency (10 Hz) envelope electrical field which triggered neural firing and the movement of mice's forepaws and whiskers [15–17]. However, to our knowledge, there is still a lack of research publication about TI study on healthy human adults now. To fill this gap, we design this study to compare the neuromodulation effects of TI and tDCS in stimulating M1 in healthy adults and analyze the online effects of TI and tDCS on brain functional connectivity in the whole brain scale. The reason for choosing tDCS as a baseline is that tDCS is a reliable and robust stimulation technique that has been widely reported in the literature. We hypothesize that TI and tDCS stimulus will both enhance functional connectivity between the M1 and related brain regions.

## 2. Methods

### 2.1. Participants

Forty healthy young adults (31 males, age: 25.97 ± 3.53 years; 9 females, age: 24.11 ± 0.93 years) were recruited in this study. The inclusion criteria are as follows: (1) all participants should be right-handed (Edinburgh Handedness Inventory; Oldfield, 1971) (2) age: 18-35 years old; and (3) no history of neurological, psychological disorder, or motor dysfunction. The exclusion criteria are as follows: (1) individuals who have contraindications with respect to the use of tDCS and (2) participants who had metallic implants/implanted electric devices were excluded. All participants have been informed of all aspects of this experiment and provided informed consent before participation. The experimental procedures were approved by the institutional review board of shanghai university of sport (102772020RT116). The Registered Clinical Trial number of this study is ChiCTR2100052866.

### 2.2. Experimental Protocol

**Figure 1:**
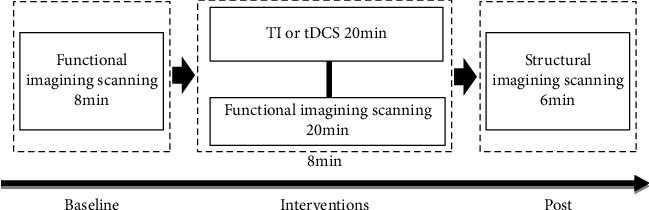
Experimental protocol. The experiment consists of baseline, intervention, and postphase. In the baseline, participants attended an 8 min functional scanning session. In the interventions phase, participants attended 20 min TI or tDCS stimulation and functional scanning simultaneously. In the post phase, participants were scanned 6 min for structural images.

This study is a randomized crossover, double-blinded design. Each participant completed two visits to accepted different types of stimulation (TI, tDCS). The time interval between the two tests was at least 48 hours. Before each test started, the stimulation target brain area of each participant was tested and located via TMS using software “STIMWEAR,” which was located in FDI's “Hot Point.” Afterward, each participant received two functional scanning sessions, one before stimulation and one during stimulation, in each visit. In the second scanning session, participants received one type of stimulation randomly. A structural scanning session was also performed in the first visit (see Figure 1).

### 2.3. TI

**Figure 2:**
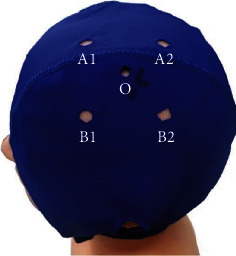
TI stimulation cap. O is the position of hot point. A1-A2 are the positions of two electrodes of one tACS channel in which the peak to peak stimulation intensity is 2 mA, and the frequency is 2000 Hz. B1-B2 are the positions of two electrodes of another tACS channel in which the peak to peak stimulation intensity is 2 mA, and the frequency is 2020 Hz. The distance between the position centers of every two electrodes is 4 cm.

“Hot Point” is the target area of TI stimulation. The representational field of right FDI was determined by a single-pulse interferential TMS stimulator (Soterix medical, New Jersey, USA). Based on the center of “hot point,” two channels of high-frequency alternating current were placed in parallel to the connection line between the eyebrow center and occipital tuberosity. We made a TI stimulation cap on which electrodes could be fixed. As shown in Figure 2, “O” is the position of “hot point”. A1, A2, B1, and B2 are the position of 4 electrodes, and the distance of four two-point pairs (A1-A2, A1-B1, B1-B2, A2-B2) is 4 cm. A1-A2 is one channel, and its frequency is 2000 Hz. B1-B2 is another channel, and its frequency is 2020 Hz. The frequency difference between these two channels is 20 Hz (2020 Hz-2000 Hz). The current intensity is 2 mA in each channel, and the total current intensity is 4 mA. The stimulation duration is 20 min, with two short periods of 30s ramp-up and ramp-down stimulation.

### 2.4. tDCS

**Figure 3:**
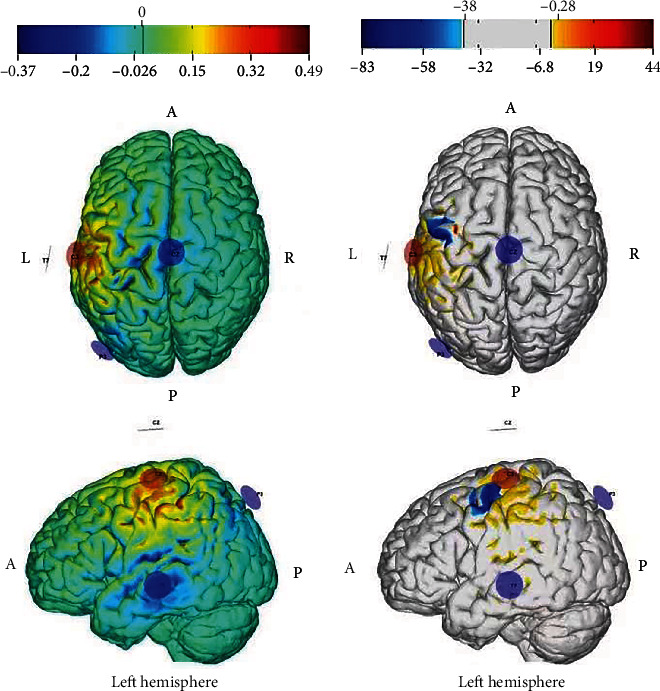
Simulated electrical field. L: left; A: anterior. The total stimulation intensity is 2 mA. The position of anodal electrode is at C3. The positions of cathodal electrodes are at CZ, T7, and P3. (a) is the normal electric field component in target region (v/m) for grey matter. (b) is the error with respect to no intervention (mv^2^/m^2^) for grey matter.

**Figure 4:**
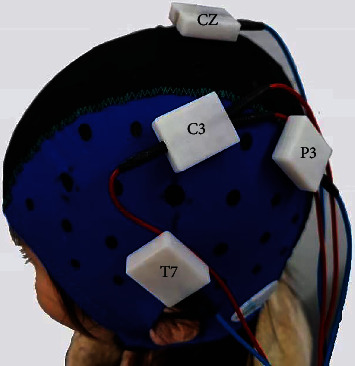
tDCS stimulation montage. Three anodal electrodes were connected to C3 silicone, and each cathodal electrode was connected to CZ, T7, and P3 silicone.

The “STIMWEAR” software provided a neuroelectric's online target editor. In this editor, we set the stimulation target area as the brain location of the right FDI, with the maximum stimulation intensity of 2 mA and the maximum stimulation electrode number of 4. The simulated result included each electrode position (10-20 electroencephalogram system) and intensity (C3: 2000 uA, P3: -774 uA, T7: -684 uA, Cz: -542 uA) (Figures 3 and 4).

The stimulator was MR-compatible DC-STIMULATOR PLUS (neuroCnn, Ilmenau, Germany). Further details of the stimulator setting can be found in the study of Esmaeilpouret al. [18]. Four rectangles MRI compatible rubber electrodes (1.5 cm × 2 cm) were used to deliver continuously direct current. The resistance of each rubber electrode was below 30 *Ω*. The stimulation duration at goal intensity was 20 min. The ramp-up and ramp-down duration were both 30s.

### 2.5. MRI Acquisition and Statistical Analysis

All participants were scanned in a 3.0 Tesla Siemens MAGNETOM Prisma whole-body MRI scanner equipped with a 64-channel head coil for radio frequency (RF) reception (Siemens, Munich, Germany). Two sessions of functional images were collected during resting state, in which all participants were asked to stare at a figure of a black cross on a white screen while relaxing, not to fall asleep, and not to think anything particularly difficult, such as mathematically calculation. The blood oxygen level-dependent (BOLD) was acquired using a gradient-echo EPI sequence (TR = 1000 ms; TE = 30 ms; FOV = 240 × 240 mm^2^; flip angle = 100°; voxel size = 3 × 3 × 3 mm^3^; 48 contiguous oblique axial slices, parallel to the AC-PC line, simultaneous multislice acquisition, three runs function scanning). The first scanning session was composed of the acquisition of a time series of 488 brain volumes, which last for 8 minutes and 8 seconds. The second session consisted of the acquisition of a time series of 1268 brain volumes, which last for 21 minutes and 8 seconds. 8 initial RF excitations were performed to achieve steady state equilibrium and were subsequently removed for each session. In the second session, the initial 30 and last 30 brain volumes were also disregarded to account for the ramp-up and ramp-down period. These factors resulted in 480 and 1200brain volumes for the first and second session, respectively, for each participant. High-resolution structural images were acquired using a 3D MP2RAGE (magnetization-prepared 2 rapid acquisition gradient echoes) pulse sequence (TR = 3130 ms; TE = 2.98 ms; flip angle = 12°; FOV = 256 × 256 mm^2^; voxel size = 1 × 1 × 1mm^3^; 166 contiguous slices).

We performed preprocessing and statistical analyses on these imaging data in DPABI (http://rfmri.org/DPABI) and SPM12 (Institute of Cognitive Neurology, London, UK. http://www.fil.ion.ucl.ac.uk), under MATLAB (Mathworks Inc., Natick, MA, USA). Default settings were chosen in the stages of early steps including preprocessing. We chose the left M1 as a seed region, extracted the time series of this seed region in each session and for each participant, then correlated with all voxels across the whole brain, and generated a correlational map for each condition. We further performed between conditions and between-group comparisons.

Activations were thresholded at *p* < 0.001, voxel-level uncorrected, and significant clusters were identified only when they also survived a *p* < 0.05, cluster-level correction for multiple comparisons, and all other clusters were filtered out. Coordinates of significant clusters peaks and subpeaks in each effect were listed in the Tables in standard MNI space. Regions were identified using the AAL atlas [19] and Brodmann templates as implemented in MRIcron (http://www.MRicro.com/MRicron).

## 3. Results

**Figure 5:**
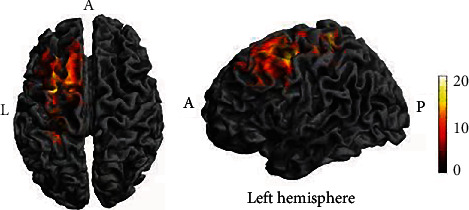
Activated brain regions in the main effect of condition. L: left; A: anterior. The color bar shows the *F* value.

A two-way repeated ANOVA (2 × 2) was used to investigate the main effects of stimulation types (TI, tDCS), stimulation phases (baseline, online stimulation), and their interaction in the brain. There was no significant main effect of stimulation types, indicating that TI and tDCS stimulation did not produce any significant difference. A significant main effect of stimulation phase was found mainly in the left precentral, paracentral lobule, SFG, SMA, SMFG, MFG, MCG (BA 4, 6, 8, 9, 32, 46), precentral, postcentral, paracentral lobule, and SPL (BA 2, 3, 4, 6, 7, 40) (Table 1 and Figure 5). TI and tDCS stimulation generated greater activation in these brain regions than the baseline. There was no significant interaction between stimulation types and phases, indicating that the TI stimulation effect was equivalent to the tDCS stimulation effect.

## 4. Discussion

To our knowledge, this is the first study to compare online effects of TI and tDCS using a seed-based whole brain functional connectivity method. Our results demonstrated that no difference existed between TI and tDCS in functional connectivity effects. Both TI and tDCS stimulation could boost functional connectivity strength between the seed M1 region and corresponding regions, and there is no difference between these two approaches.

### 4.1. Effects of TI and tDCS

Between-group analysis, our results observed that no difference between tDCS and TI on seed-based functional connectivity. This finding was also demonstrated within-group analyses. Comparing to the prestimulation, online TI and tDCS both increase the ipsilateral functional connectivity in MFG, MSFG, SFG, and SMA. To our knowledge, there is no record of TI effects in healthy human beings. Previous studies investigated the tDCS effect on resting-state functional connectivity, and their results are similar to our results to some extent. In the study of Sehm et al. [11], they found that tDCS induced online and afterward effects on seed-based functional connectivity, and after the termination of the intervention, and induced an increase in functional connectivity within ipsilateral hemispheric. Moreover, the results of Polania's study [14] showed that tDCS induced functional connectivity increased in ipsilateral M1, premotor cortex, and sensorimotor area. However, in contrast to Polania's study, the functional connectivity increased area in our study only lie in MFG, MSFG, SFG, and SMA, which are involved in motor planning and motor learning. This difference may cause by the stimulation montage that we choose. Based on the current field simulation, we used multifocal stimulation montage to optimize the stimulation effects. But in Polania's study, they used electrodes on a 5 × 7 cm area, which covered many brain regions, to deliver current to the scalp. A previous study showed that the size of the electrodes is a factor in modulating the stimulation effect [20]. In the study of Ho [21], they found that stimulated with a larger size induced a cumulative enhance in cortical excitability, but not a smaller electrode.

The tDCS and TI induced seed-based functional connectivity increased with MFG, MSFG, SFG, and SMA areas. Those areas may be related to motor function. In the studyof Rosse [22], they demonstrated that the functional connectivity between premotor cortex and M1 has a negative relationship with resting motor threshold in M1. The lower resting motor threshold in M1 means higher cortical excitability. Thus, the study of Rosse et al. provided support to the notion that the functional connectivity between premotor cortex and M1 has a positive relationship with cortical excitability. It means that the increasing cortical excitability would enhance human performance [23–26]. Cogiamanian's study reported that anodal tDCS improve muscle endurance by improving cortical excitability [27]. Therefore, TI and tDCS induce the modulation coupling of the functional network, which may enhance human performance.

### 4.2. Comparing the Neurophysiological Mechanism TI and tDCS

Although TI and tDCS online effects are similar, the underlying mechanisms of these two types of stimulation may be different. In tDCS, anodal tDCS may restrain the inhibitory synaptic to increase the resting-state functional connectivity. Previous studies showed that anodal tDCS decreased the gamma-aminobutyric acid (GABA) concentration [28–30]. The GABA concentration was negatively correlated with the strength of resting-state functional connectivity within the motor network [28, 29]. In the study of Bachtiar et al. [28] and Stagg et al. [29], they both demonstrated that the anodal tDCS decreased GABA concentration and increases functional connectivity in the stimulated motor cortex. In the TI, it may modulate the brain oscillation to change the resting-state functional connectivity. Grossman's study [17] showed that TI impacts mice's brain function via a temporal interference field, which was produced by a high-frequency field with slightly different frequencies. The effect of a lower frequency interference field is similar to lower frequency transcranial alternating current stimulation, which mainly depends on the current frequency [31]. In previous studies, 20 Hz-tACS stimulation was applied over M1 that modulation the brain oscillation to increase the excitability of M1 and optimize the participant's performance [31–33]. In our study, we used an interference frequency of 20 Hz (2020 Hz minus 2020 Hz). 20 Hz-TI may change the brain oscillation and increase the resting-state functional connectivity between M1 and secondary motor cortex to improve human performance. Therefore, TI and tDCS may modulate brain function via different neurophysiology mechanisms.

### 4.3. Limitation

Although we carefully designed the study, there are still many caveats. Overcoming those caveats may be helpful for future studies. (1) The stimulation intensity is too low to induce sufficient effects. TI applied high-frequency current intensity attenuate rapidly in human deep tissue [17]. In our study, the stimulation intensity is 2 mA. The intensity is not high enough to induce the interaction effect of intervention by time. Therefore, in the future study, it can apply much higher current intensity within the ethical limits. (2) It is a lack of a tACS group. TI may impact brain function by modulating brain oscillation, which is similar to tACS. Adding a tACS group may be better to compare the effects and better understand the neurophysiological mechanisms of TI, tDCS, and tACS. (3) In the current study, we did not provide an effective method in simulating the charge density of TI. In the future, TI electric field could be simulated to confirm whether the charge density is equal in different stimulation montages.(4) Forty-eight hours may not be long enough to wash out the posteffect, and longer time intervals are recommended for future studies. (5) The current study did not investigate the long-term effect of TI and tDCS. It could be examined in the future study.

## 5. Conclusion

TI and tDCS both increased resting-state functional connectivity between M1 and secondary motor cortex (premotor cortex and supplementary motor cortex), and the enhancement of functional connectivity may be related to motor functions.

## Figures and Tables

**Table 1:** Activated brain regions in the functional connectivity analysis.

Contrast	Cluster regions	*p*	Cluster size	Peak *Z* value	Peak MNI coordinates		
					*X*	*Y*	*Z*
Main effect of condition	L: precentral, paracentral lobule, SFG, SMA, SMFG, MFG, MCG	<0.001	1078	4.4	-33	-1	53
	L: precentral, postcentral, paracentral lobule, SPL	<0.001	218	4.13	-27	-37	65

L: left; MCG: middle cingulate gyrus; MFG: middle frontal gyrus; SMA: supplementary motor area; SFG: superior frontal gyrus; SMFG: superior medial frontal gyrus; SPL: superior parietal lobule.

## Data Availability

All experimental data, together with relevant analysis scripts and files, are available upon request from the corresponding author (e-mail: jzhuang255@163.com).
